# Common solar wind drivers behind magnetic storm–magnetospheric substorm dependency

**DOI:** 10.1038/s41598-018-35250-5

**Published:** 2018-11-19

**Authors:** Jakob Runge, Georgios Balasis, Ioannis A. Daglis, Constantinos Papadimitriou, Reik V. Donner

**Affiliations:** 1 German Aerospace Center, Institute of Data Science, 07745 Jena, Germany; 2 National Observatory of Athens, Institute for Astronomy, Astrophysics, Space Applications and Remote Sensing, Penteli, 15236 Athens Greece; 3 National and Kapodistrian University of Athens, Department of Physics, 15784 Athens, Greece; 4 Potsdam Institute for Climate Impact Research, 14473 Potsdam, Germany; 5 Imperial College, Grantham Institute, London, SW7 2AZ United Kingdom; 6 Magdeburg-- Stendal University of Applied Sciences, 39114 Magdeburg, Germany

## Abstract

The dynamical relationship between magnetic storms and magnetospheric substorms is one of the most controversial issues of contemporary space research. Here, we address this issue through a causal inference approach to two corresponding indices in conjunction with several relevant solar wind variables. We find that the vertical component of the interplanetary magnetic field is the strongest and common driver of both storms and substorms. Further, our results suggest, at least based on the analyzed indices, that there is no statistical evidence for a direct or indirect dependency between substorms and storms and their statistical association can be explained by the common solar drivers. Given the powerful statistical tests we performed (by simultaneously taking into account time series of indices and solar wind variables), a physical mechanism through which substorms directly or indirectly drive storms or vice versa is, therefore, unlikely.

## Introduction

The identification of spurious associations and potentially causal relationships is key to an improved process-based understanding of various geoscientific processes. Specifically in magnetospheric physics, the understanding of the relationship between magnetic storms and magnetospheric substorms as a part of the solar wind–magnetosphere system is of paramount importance for the development of numerical simulation models of the magnetosphere^1^. In particular, the existence and directionality of the storm – substorm interaction is one of the most controversial aspects of magnetospheric dynamics^2^. The original concept of storms being the cumulative result of successive substorms put forward by Akasofu in 1961^3^ has been disputed in subsequent analyses^2,4,5^. While several model-based studies have shown a distinct impact of substorm injections on the storm-time ring current enhancement^6–8^, other studies have suggested that the ring current buildup could in principle be directly driven by the solar wind electric field^9,10^. In this case, magnetospheric substorms do not drive magnetic storms and the two phenomena are independent and share a common cause–the southward interplanetary magnetic field (IMF) driver^11^.

Recent work (see for instance the review by Balasis *et al*.^12^) points to a considerable importance of entropy-based measures for identifying and quantifying linear and nonlinear interdependencies between different geophysical variables, variability at different scales, and other characteristics. Time series analyses based on information-theoretic measures have been used to shed light on the storm-substorm interaction^13^ and the solar wind drivers of the outer radiation belt^14^ through the general perspective of quantifying information transfer, including linear and nonlinear mechanisms. In particular, DeMichelis *et al*.^13^ applied a bivariate transfer entropy^15^ (bivTE) analysis to the geomagnetic activity indices AL and SYM-H. SYM-H is the high-resolution (1-min) version of the hourly Disturbance storm-time (Dst) index, which is used as a proxy of magnetospheric ring current strength and, thus, as a measure of magnetic storm intensity. AL belongs to the set of the 1-min Auroral Electrojet indices (AE, AL, AU and AO) and is used to determine the onset of the substorm growth phase^16^. DeMichelis *et al*. suggested that information flow from AL to SYM-H dominates in the case of small geomagnetic disturbances, while the reverse situation is observed in presence of strong geomagnetic disturbances.

However, bivariate measures such as mutual information (MI) or bivTE do not allow to exclude the very frequent influence of other variables as common drivers, rendering MI and bivTE associations spurious. Multivariate extensions of TE, on the other hand, are severely limited because their estimators don’t work well in high dimensions^17^. In the present study, we contrast bivariate measures with a directional, multivariate information-theoretic causality measure based on low-dimensionally estimated graphical models^17–19^. This multivariate measure for the influence of a subprocess *X* of a system on another subprocess *Y* is called *information transfer to Y* (ITY) and allows for more powerful tests on the absence or potential presence of a causal relationship, which is crucial for developing a better “mechanistic” understanding of the governing processes.

Here, we investigate time series of various solar wind parameters including the IMF’s magnitude *B* and vertical component *B*_*Z*_, its velocity *V*_*SW*_ and dynamic pressure *P*_*dyn*_, as well as the AL and SYM-H indices. We focus on data from 2001, near a solar activity maximum. Our goal is to clarify whether substorm activity could causally drive storm dynamics or – on the contrary – whether solar wind variables can explain the statistical associations between storm and substorm activity.

## Results

### Data

The present study is focused on the year 2001, a year with very strong solar activity, and data on solar wind parameters as well as geomagnetic activity indices. As possible solar driving factors we include only those quantities with at least a measurable statistical dependency (mutual information) with either AL or SYM-H. These are *B*, *B*_*Z*_, *V*_*SW*_, and *P*_*dyn*_. The original one-minutely data were aggregated to a 20-minute time resolution by averaging over non-overlapping 20-minute blocks. This resolution was selected based on iterative tests to obtain a compromise between resolving time lags and still keeping the computational load and multiple testing problems low. Additionally, DeMichelis *et al*.^13^ found, on average, a net information flow from AL to SYM-H attaining its maximum at a typical time delay of about 1 h which is well resolved with our chosen time resolution.

**Figure 1:**
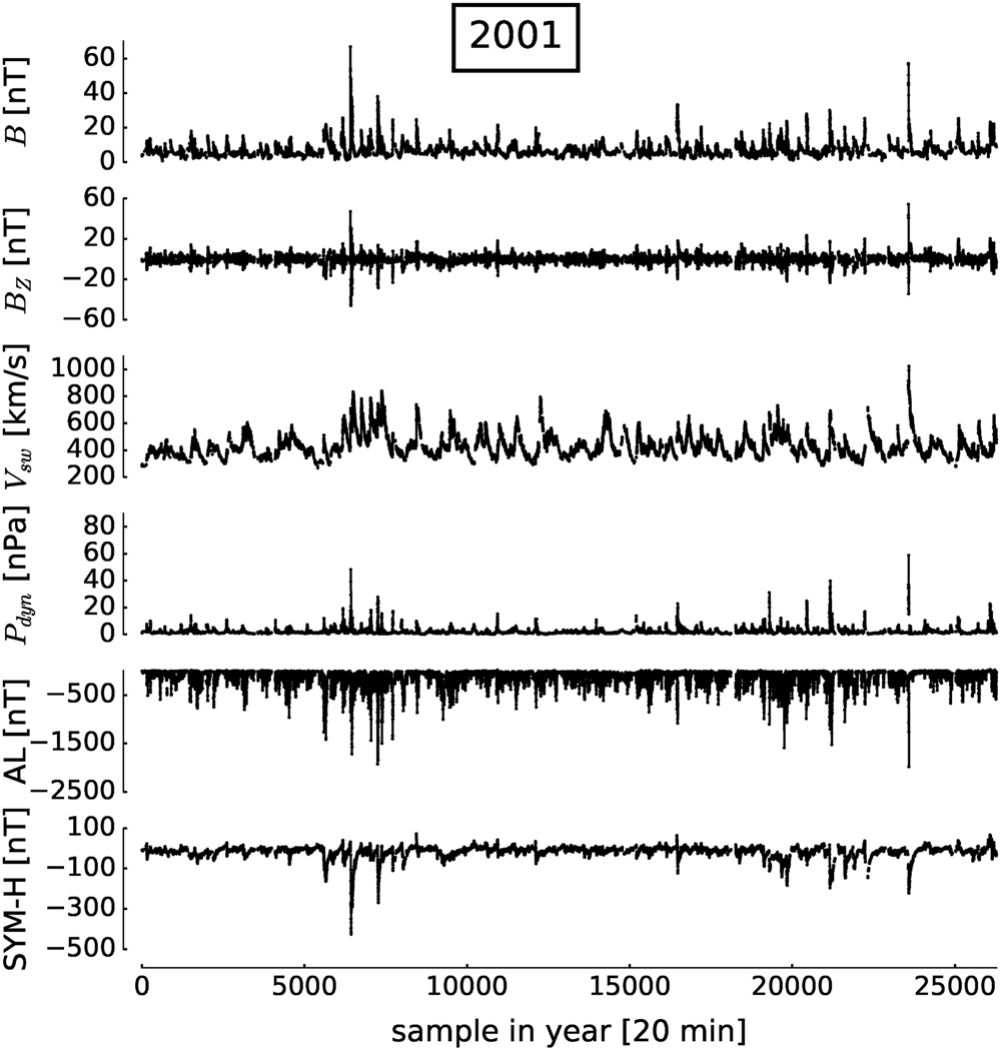
Time series of solar and magnetospheric variables for 2001. Time points with missing values in any of the variables are excluded from the analysis, taking lags into account. Clearly, there is strong solar and magnetospheric activity, in Supplementary Table S1 we classify storms into moderate, intense, and super-storm events.

As solar wind time series inevitably contain missing values due to satellite failures, in the aggregation we masked samples for 20 min periods with more than 50% missing values. We also accounted for masked samples in the lagged analyses (up to *τ*_*max*_ = 6 × 20 min) to avoid a selection bias. This leads to 18,384 non-masked 20-min samples instead of about 26,000 samples for the whole year. No further pre-processing was applied. Figure 1 shows the corresponding time series.

### Mutual information and bivariate transfer entropy analysis

This study aims to shed light on the possible existence of a driver-response relationship between storms and substorms, which is a reflection of the dynamic processes within the coupled solar wind-magnetosphere-ionosphere system. Because there has been accumulating evidence that the involved interrelations are of a nonlinear nature^20–26^ and very long data series are available, we employ a non-parametric (model-free) approach here. Information theory provides a genuine framework for the model-free study of couplings among time series. Here we invoke three information-theoretic measures with increasing power to detect spurious dependencies due to autocorrelation, common drivers or indirect relationships.

The first and simplest association measure applying information theory to time series is the lagged (cross-)mutual information^27^ given by

1 $${I}_{XY}^{{\rm{MI}}}(\tau )=I({X}_{t-\tau };{Y}_{t})=H({Y}_{t})-H({Y}_{t}|{X}_{t-\tau }),$$

using Shannon entropies $H(X)=-\,\int \,p(x)\,\mathrm{ln}\,p(x)dx$ (correspondingly for conditional entropies) in units of *nats* with the natural logarithm as a base. For *τ* > 0, MI measures the information in the past of *X* that is contained in the present of *Y*. The weaknesses of MI as a measure of information transfer have been discussed early on, most notably by Schreiber^15^. A first step to arrive at a directional notion of information transfer is to exclude information from the past of *Y*. Implementing this idea, Schreiber introduced the *transfer entropy* (TE)^15^ between two variables, which is the information-theoretic analogue of Granger causality and can be defined in a lag-specific variant as

2 $${I}_{X\to Y}^{{\rm{bivTE}}}(\tau )=I({X}_{t-\tau };{Y}_{t}|{Y}_{t-1})$$

based on the *conditional mutual information*. To estimate all CMIs in this study, we use an advanced nearest-neighbor estimator^28,29^ that is most suitable for variables with a continuous range of values (details in Methods).

**Figure 2:**
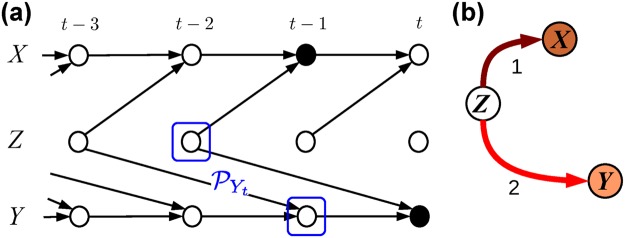
Example of causal interactions in a three-variable process. (**a**) Time series graph^30^ which encodes the spatio-temporal dependencies. The set of *parents* ${{\mathscr{P}}}_{{Y}_{t}}$ (blue boxes) separates *Y*_*t*_ from the past of the whole process ${{\bf{X}}}_{t}^{-}\backslash {{\mathscr{P}}}_{{Y}_{t}}$, which implies conditional independence (Markov property) and is used in the algorithm to estimate the graph^17,19^. (**b**) Process graph, which aggregates the information in the time series graph for better visualization (labels denote the lags, link and node colors denote the cross- and auto-coupling strength).

However, bivTE can yield spurious results if more than two processes are interacting: For the interaction example in Fig. 2(b) both the MI *I*(*X*_*t*−1_;*Y*_*t*_) and the TE *I*(*X*_*t*−1_;*Y*_*t*_|*Y*_*t*−1_) are larger than zero due to the common driver *Z*, even though no direct or indirect physical mechanism exists by which *X* drives *Y* or vice versa. The detailed time-resolved graph in Fig. 2(a) shows that, *X*_*t*−1_ and *Y*_*t*_ are *not* independent given only the past of *Y* or only the common driver *Z*_*t*−2_ as a condition. Rather, in order to unveil the spurious dependency, the CMI must be conditioned on both *Y*_*t*−1_ and *Z*_*t*−2_ to exclude all causal paths connecting *X*_*t*−1_ and *Y*_*t*_ (see ref.^30^ for a definition of causal paths).

**Figure 3:**
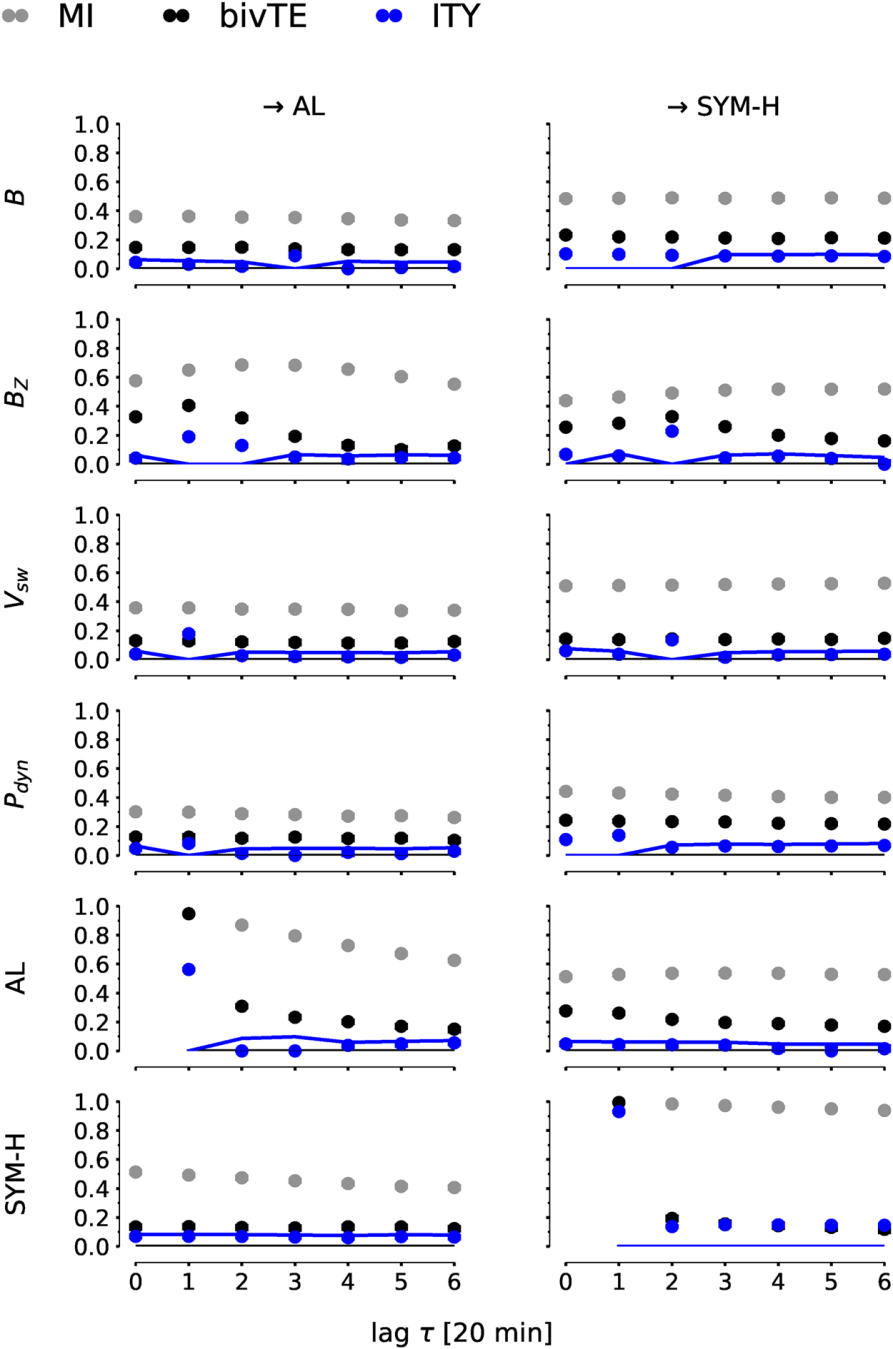
Lag functions of information-transfer measures. The lag functions were estimated with nearest-neighbor CMI estimation parameter *k* = 50^28,29^. For example, the panel *B*_*Z*_ → AL shows the lag function $I({B}_{Z,t-\tau };{{\rm{AL}}}_{t}|\ldots )$ of MI (Eq. (1), gray), bivTE excluding the past lag of AL (Eq. (2), black), and the multivariate ITY (Eq. (3), blue) conditioning out the influence also of other variables with the parents ${\mathscr{P}}$ given in Table 1. All (C)MI values have been rescaled to the (partial) correlation scale via $I\to \sqrt{1-{e}^{-2I}}\in [0,1]$^27^. For ITY, the solid line marks the significance threshold. MI and bivTE are clearly significant for a large range of lags. Confidence intervals (errorbars) are mostly smaller than the dots. MI and bivTE with their broad peaks clearly provide no precise information about relevant drivers and coupling delays. On the other hand, ITY features large values only at few selected lags. In Supplementary Fig. S1 we show that these results are robust for further method parameters and storm indices.

In Fig. 3 we investigate bivariate MI and bivTE lag functions of all considered solar variables with AL and SYM-H, including the interaction between these two. We restrict the maximum time delay to *τ*_*max*_ = 6 × 20 min. For example, the panel *B*_*Z*_ → AL shows the lag function *I*(*B*_*Z*,*t*−*τ*_; AL_*t*_) of MI [Eq. (1), gray] and *I*(*B*_*Z*,*t*−*τ*_; AL_*t*_|AL_*t*−1_) of bivTE excluding the past lag of AL [Eq. (2), black]. The multivariate ITY [Eq. (3), blue] is discussed in the next section. The solid line marks the significance threshold. All (C)MI values have been rescaled to the (partial) correlation scale via $I\to \sqrt{1-{e}^{-2I}}\in [0,1]$^27^ and rescaled values above 0.4 can, thus, be considered as moderate to strong. In the tables (Table 1 in main article and Supplementary Tables S2–S4), on the other hand, the CMI values are given in *nats*.

**Table 1:** Multivariate information-theoretic analysis of dependency between AL and SYM-H.

Parents of SYM-H (*k* = 50)			
Parent (*τ* [20 min.])	*I*_Par_ → _SYM-H_	*I*_AL_ → _SYM-H_	*p*-value
No conds.		0.1634	<10^−2^
SYM-H (−1)	2.2092	0.0353	<10^−2^
+*B*_*Z*_ (−2)	0.0572	0.0089	<10^−2^
+*P*_*dyn*_ (−1)	0.0164	0.0022	<10^−2^
+*V*_*sw*_ (−2)	0.0094	0.0010	0.635
**Parents of AL (*k*** = **50)**			
**Parent (** ***τ*** **[20 min.])**	***I***_**Par**_ → _**AL**_	***I***_**SYM-H**_ → _**AL**_	***p*** **-value**
No conds.		0.1388	<10^−2^
AL (−1)	1.1426	0.0094	<10^−2^
+*B*_*Z*_ (−1)	0.0901	0.0059	<10^−2^
+AL (−3)	0.0159	0.0056	<10^−2^
+*V*_*sw*_ (−1)	0.0188	0.0034	0.035
+*B* (−3)	0.0057	0.0030	0.070
+*B*_*Z*_ (−2)	0.0077		
+*P*_*dyn*_ (−1)	0.0034		

**Figure 4:**
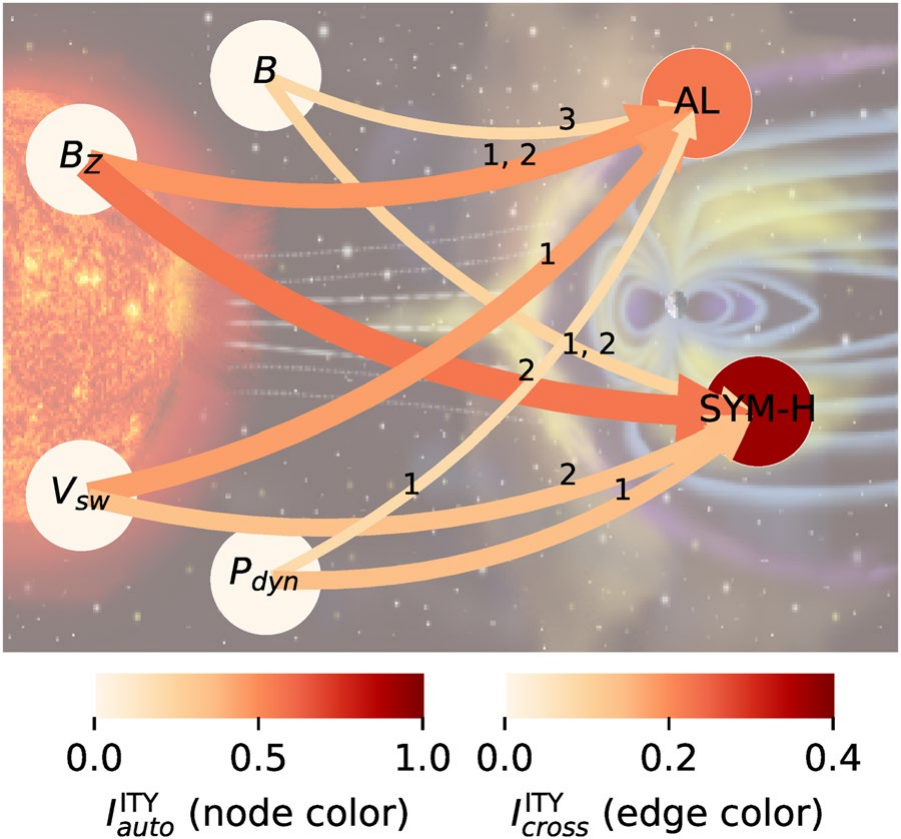
Graph based on significant ITY values at the 95% level in Fig. 3. Edges correspond to directional lagged links, and the labels indicate their lags. If more than one lag is significant, they are listed in the order of their strength. The edge color and width indicate the value at the lag with the largest ITY. The node color depicts the strength of the lag-1 auto-dependency for AL and SYM-H. Note that the weak ITY value in *B*_*Z*_ → SYM-H is likely due to *B*_*Z*_ occurring with two neighboring lags in the parents of SYM-H, which reduces the information transfer of either of them. In Supplementary Fig. S2 we show the robustness of these results using a different CMI estimation parameter and another substorm index.

The first column lists the iteratively tested conditions [variable (lag)] regarding the links AL → SYM-H (top) and SYM-H → AL (bottom), both at lag 1. In each step the set of conditions with the highest CMIs in the preceding step is chosen. In the present analysis these sets always contained the conditions of the previous iteration (which is not necessarily the case, see ref.^30^). The second column gives the CMI value of the added condition. The third column gives the CMI value for the substorm – storm link at lag 1 using the conditions in this step and the last column its *p*-value. The *p*-values are computed from a block-shuffle ensemble of 200 surrogates (see Methods). The storm – substorm link vanishes in both years with a *p*-value larger than 0.05 after few solar drivers have been taken into account. The full list of parents in the first column is then used in a second step to estimate ITY (see lag functions in Fig. 3 and graphs in Fig. 4). The nearest-neighbor parameter was set to *k* = 50, the analysis with very similar results for *k* = 100 and another substorm index (AE) is shown in Supplementary Figs S1 and S2 and Tables S2–S4. Here all CMI values are measured in *nats* and are not rescaled to the partial correlation scale as in the figures.

In Fig. 3, MI lag functions (grey) show large values for all possible driver variables. Here the peak of the MI lag function is often shifted compared to bivTE (black). Such an effect can arise from strong autocorrelations as studied in ref.^31^. Overall, the bivariate TE has sharper peaks than MI. *B*_*Z*_ clearly is the strongest driver of both AL and SYM-H, and all other drivers are comparably weak (except for the auto-dependencies in panels AL → AL and SYM-H → SYM-H). The reason for this behavior is that some MI values are ‘inflated’, again, due to strong autocorrelations^18^, especially *V*_*sw*_ is strongly auto-dependent. This makes MI values and the peak of MI lag functions hard to interpret.

The interactions AL → SYM-H and SYM-H → AL have been studied in ref.^13^ where a relationship from substorms towards storms was found with a binning estimator of bivTE. Our results reproduce this finding with a nearest-neighbor estimator^28,29^. The other direction, from storms to substorms, is not very significant here. Note that values at lag *τ* = 0 min cannot be interpreted in a directional sense in our analysis.

### Multivariate information-theoretic causality analysis

Mutual information and bivariate information-theoretic measures, such as MI and bivTE, cannot account for common drivers and indirect transitive relationships. As illustrated in Fig. 2(b), a common driver (*Z*) can lead to a spurious association, either linear or nonlinear, between *X* and *Y*. The complex multivariate causal interaction structure can be captured with the concept of a *time series graph*^32,33^ as shown in Fig. 2(a), originating from the theory of graphical models. As further defined in ref.^30^, each node in a time series graph represents a subprocess at a certain time. Past nodes at *t*′ < *t* have a link towards a subprocess at time *t* if and only if they are not independent conditionally on the past of the whole process. In this graph the parents ${{\mathscr{P}}}_{\cdot }$ of a variable are given by all nodes with an arrow towards it (blue boxes in Fig. 2(a)).

While these parents could be estimated by testing the CMI between each *X*_*t*−*τ*_ and *Y*_*t*_ conditional on all other lagged variables, this approach, similar to multivariate or conditional TE, does not work well due to its high dimensionality^17^ leading to weak statistical power and many false positives. In ref.^19^ an efficient algorithm for the estimation of the parents of a variable *Y* (further details in Methods) is detailed. In a second stage we use the estimated set of parents to measure the *information transfer to Y* (ITY)^18^ for all lagged variables *X*_*t*−*τ*_ (including the parents)

3 $${I}_{X\to Y}^{{\rm{ITY}}}(\tau )=I({X}_{t-\tau };{Y}_{t}|{{\mathscr{P}}}_{{Y}_{t}}),$$

which will be zero if and only if *X*_*t*−*τ*_ and *Y*_*t*_ are independent *conditionally on* ${{\mathscr{P}}}_{{Y}_{t}}$. Unfortunately, no analytical results exist on the finite-sample distribution of the nearest-neighbor estimator under the null hypothesis of conditional independence. For significance testing, we use a block-shuffle surrogate test here following refs.^34^ and^35^ as described in Methods. The algorithm was run with maximum lag *τ*_*max*_ = 6 × 20 min as before. We assess significance at the 95% level.

Table 1 shows iteration steps with the selected conditions and the conditional mutual information (CMI) values and significance of the AL → SYM-H and SYM-H → AL links in each step. The AL → SYM-H link becomes non-significant using the condition set (SYM-H(*t* − 1), *B*_*Z*_(*t* − 2), *P*_*dyn*_(*t* − 1), *V*_*sw*_(*t* − 2)). This implies that these solar drivers can explain the spurious link AL → SYM-H at a lag of 20 min. Note that this set is only a sufficient explanatory set and other drivers might also induce this spurious association. Also the much weaker link SYM-H → AL becomes non-significant after including few solar drivers (*B*_*Z*_, *V*_*sw*_, *B*, at different lags).

The ITY estimates with these parents are shown in Fig. 3 (blue markers). ITY now accounts for autocorrelation in the driven variable (like bivTE), but additionally for the influence of the other parents as common drivers or indirect mediators^36^. Now the ITY lag functions are peaked and significant (markers above solid line) only at a few selected lags.

Figure 4 visualizes the significant drivers of AL and SYM-H in a process graph as in Fig. 2(b). Edges correspond to directional lagged links, and the labels indicate their lags. If more than one lag is significant, they are listed in the order of their strength. Both, the edge color and width, indicate the value at the lag with the largest ITY. The node color depicts the strength of the lag-1 auto-dependency for AL and SYM-H. Note that the weak ITY value in *B*_*Z*_ → SYM-H is due to *B*_*Z*_ occurring with two neighboring lags in the parents of SYM-H, which reduces the information transfer of either of them.

In conjunction with some further robustness studies for another substorm index and other method parameters (Supplementary Figs. S1 and S2), our major results can be summarized as follows: The main drivers of substorms as measured by AL are *B*_*Z*_ and *V*_*SW*_. These also drive storms as measured by SYM-H. *P*_*dyn*_ and especially *B* are less robustly related to both storms and substorms. Regarding time lags, the AL index responds to *B*_*Z*_ at a lag ≈ 20–40 min, while the lags with the weaker other drivers are less robust (see Supplementary Figs. S1 and S2). The SYM-H index responds to *B*_*Z*_ at a lag ≈ 40 min, to *V*_*SW*_ at 40 min, to *P*_*dyn*_ at 20 min, and rather weakly with non-robust lags to *B*.

Regarding the previously found link AL → SYM-H^13^, we find that mainly *B*_*Z*_ and to a lesser degree *V*_*SW*_ and *P*_*dyn*_ are sufficient to explain this statistical association. These results are also verified by applying the same tools to an AE - SYM-H analysis and for other estimation parameters (see Supplementary Figs. S1 and S2 and Tables S2–S4). Thus, we find that there is no direct or indirect transfer of information AL → SYM-H or SYM-H → AL.

## Discussion

DeMichelis *et al*.^13^ investigated the transfer of information between substorms and storms by means of a bivariate transfer entropy analysis of AL and SYM-H time series from 1981 (near solar maximum). They found a significant information flow from substorms to storms attaining its maximum with a typical time delay of about 1 h and suggested that the direction of information flow between substorms and storms depends on the global magnetospheric activity level. Our analysis goes beyond the study of ref.^13^ by utilizing a directional, multivariate information-theoretic causality measure that simultaneously takes into account solar wind variables and geomagnetic indices data, allowing for more powerful statistical tests on the absence or potential presence of a causal relationship between substorms and storms.

Our secondary finding that the main drivers of substorms (as measured by AL) and storms (as measured by SYM-H) are *B*_*Z*_ and *V*_*SW*_ is consistent with the fact that the energy transfer from the solar wind to the magnetosphere is proportional to *B*_*Z*_ and *V*_*SW*_. *P*_*dyn*_ and especially *B* are less relevant for both storms and substorms. We conclude that these directed information transfers constitute robust interrelationships between solar wind parameters and dynamic processes in the magnetosphere. These findings confirm earlier studies on solar wind drivers and their storm and substorm manifestations, including variations of the indices SYM-H and AL (refs.^37,38^ and refs. therein).

Johnson *et al*.^39^ recently showed that the transfer of information from *V*_*SW*_ or *VB*_*south*_ (where *VB*_*south*_ is *V*_*SW*_ × southward IMF *B*_*Z*_) to Dst (similar to SYM-H but with a lower resolution) lasts more than 100 hours, which may correspond to the long time scale of the ring current decay (e.g., ref.^40^). We have also considered similar time scales when having analyzed *VB*_*south*_ and Dst for another study focusing solely on storms^41^. Here, we analyze 1 minute-resolution indices and solar wind data in order to look at shorter time scales that cover lags up to 2 hours, focusing both on storms and substorms. The finding of IMF *B*_*Z*_ and *V*_*SW*_ as the drivers of storms are consistent with Johnson *et al*.^39^, which also used information theoretic tools (transfer entropy and cumulant based analysis) in their analysis.

Our most important finding is that our iterative causal discovery algorithm analysis suggests that mainly *B*_*Z*_, and to a lesser degree *V*_*SW*_ and *P*_*dyn*_ are sufficient to explain the previously found link AL → SYM-H^13^. Thus, we find no statistical evidence for a link AL → SYM-H. We also find no link SYM-H → AL and these results are robust also for another substorm index (AE) and for other estimation parameters. The results by Iyemori and Rao^42^ supported the idea that the geomagnetic storms and substorms are independent processes; that is, the ring-current development is not the result of the frequent occurrence of substorms, but that of enhanced convection caused by the large southward IMF. Although some later studies^43,44^, based on in-situ observations, have shown that the contribution of ion injections to the ring current energy gain is substantial, our results do not favor the role of substorms in the enhancement of the storm-time ring current through accumulative ion injections during consecutive substorms, in agreement with the earlier studies by Iyemori and Rao^42^. A possible reason for the absence of information transfer from AL → SYM-H might be that not all ion injections to the storm-time ring current are reflected in the AL variations. A recent study^45^ showed that small-scale injections are not captured by AL. Another study^46^ showed that low- and high-energy protons vary in quite different ways on storm-time timescales and accordingly suggested that the relation between ion injections and ring current growth may be more complicated than previously perceived. In summary, it is possible that substorms are required for the particle injection to the ring current [e.g. seen in Energetic Neutral Atom imaging] but not sufficient (since non-storm substorms appear to lead to no intensification of the ring current) and strong convection is also required. This remains a debate.

Before concluding, let us discuss the methodological limitations pertaining to such a statistical causality analysis. The presence of significant links in our analysis can be called causal only with respect to the included set of variables. Non-observed variables can still be the cause of a link here and the obtained links should, therefore, serve more as an hypothesis for further studies that include more possible explanatory variables. From a theoretical standpoint, a more robust finding is that of the absence of a link: if there exists no statistical evidence for a dependency between two variables, a physical mechanism between the two is unlikely. Hence, the non-significance of direct or indirect dependencies between the commonly used AL and SYM-H indices leads us to the conclusion that there exists no physical mechanism by which perturbations in substorms are transported to storms or vice versa. However, from a practical standpoint in the general context of the limitations associated with every statistical information quantity, below we summarize possible deficiencies and/or weaknesses that may accompany the application of ITY, even though we consider them rather insignificant or of low probability to occur for the present study.

Firstly, the information measure might not capture the dependency. We should note that our information-theoretic approach allows to take into account almost any type of nonlinear relationship, both in excluding it as a common driver, and also in detecting it. This is in contrast to linear correlation or linear Granger causality studies. The price for this “generality” is lower statistical power: For a particular dependency, the more general CMI will have less power compared to a measure that is optimized for this type of dependency, for example correlation for linear dependencies. Weaker power means that weaker dependencies might not be detected for small sample sizes, especially for high-dimensional conditions^30^. Our method is designed to avoid high-dimensionality by an iterative approach (especially compared to multivariate TE) and has demonstrated high power in numerical experiments^30^. Additionally, here we have a very large sample size, leading us to the conclusion that if there is a dependency, it must be very weak. Also, our major finding is robust when using other estimation parameters.

Secondly, we analyzed the whole year 2001 to obtain a sufficiently large sample size. Possibly, a causal relationship is present only during shorter periods and absent in other periods, which would be difficult to assess given too short sample sizes and the length of characteristic time scales of the processes.

Thirdly, the physical mechanism might be present mostly during the missing values excluded in the analysis. If satellite failures are indeed strongly related with the hypothesized mechanism, this would imply a non-avoidable selection bias in our analysis.

Lastly, the indices have serious limitations as to their ability of monitoring a particular current system: (1) They are scalars and may be insufficient to deduce a 2D current system; (2) The indices are derived from a very limited number of stations and as such are subject to a number of artifacts and limitations; (3) The ground perturbations are due to all currents – near and far. In summary, AL is a limited measure of the 2D westward electrojet distribution (intensity, structure and dynamics) and SYM-H is a limited measure of the ring current (intensity, structure and dynamics)^47^. Here we tested two kinds of indices for substorms (AL and AE) and got robust results.

These limitations (generality-power trade-off, missing values, proxy data quality) apply to any statistical coupling analysis. The main shortcoming of previous approaches based on bivariate measures is that these did not take into account possible common drivers, hence weakening a possible causal interpretation. Multivariate approaches have a stronger causal interpretation at the cost of weaker detection power due to higher dimensionality, which our method alleviates as much as possible. In light of these qualifications, we conclude that a direct or indirect physical mechanism by which substorms drive storms or vice versa is unlikely.

## Conclusions

There has been only one study so far that utilized information theory tools to study the storm-substorm relation. De Michelis *et al*.^13^ used the bivariate measures of delayed mutual information and transfer entropy to analyze SYM-H and AL indices from a year near solar maximum (1981). Their findings suggested that information flow from AL to SYM-H dominates in the case of weak geomagnetic disturbances, while the reverse situation is observed in the presence of strong geomagnetic disturbances. The present study goes beyond the analysis performed by De Michelis *et al*.^13^ by contrasting bivariate measures with a directional, multivariate information-theoretic causality measure based on low-dimensionally estimated graphical models. This multivariate measure is called information transfer to *Y* and allows for more powerful tests on the absence or potential presence of a causal relationship, which is crucial for developing a better “mechanistic” understanding of the governing processes. Thus, we are able to simultaneously handle SYM-H and AL indices along with the magnetospheric activity solar wind variables including the IMF’s magnitude *B* and vertical component *B*_*Z*_, its velocity *V*_*SW*_ and dynamic pressure *P*_*dyn*_ using an information measure technique. This is the first time, to our knowledge, that the variations of the various parameters describing the input and output of the solar wind – magnetosphere system are treated all together by a causality measure that is capable to identify information flow between all these parameters. We conclude on non-significant direct or indirect dependencies between AL and SYM-H indices, and therefore, between substorms and storms, which means that the previously applied bivariate measures were not able to accurately depict or resolve the interdependencies between the system’s parameters.

Additionally, a secondary conclusion of our study is that we are able to confirm earlier results about the solar wind drivers of storms and substorms (i.e., *B*_*Z*_) from a different point of view, utilizing the modern and versatile toolbox of information theory. We have achieved this, for the first time, by considering the solar wind-magnetosphere system as a whole and applying a multivariate information-theoretic approach able to simultaneously handle the system’s input (solar wind drivers) and output (magnetospheric activity indicators) in contrast to several previous important but distinct studies, where the bulk of information on the solar wind driver of the magnetosphere has been accumulated [e.g. refs.^37,38^]. This demonstrates the great potential that the application of information theory may have to treat space physics problems, where vast amounts of related datasets are continuously accumulated either from spaceborne or ground-based measurements. Moreover, in the light of our findings, the application of the multivariate causality measure of ITY was able to explain the spurious link AL → SYM-H found previously by bivariate causality measures^13^ simply by the variations of the solar wind drivers.

The results of this study contribute to the ongoing debate of the storm-substorm relationship and to the debate of plasma injection to the inner magnetosphere. For example, Angelopoulos *et al*.^48^ concluded that bursty-bulk flows (BBFs) are sufficient to account for all the energy deposition in the ionosphere and inner magnetosphere. However, Ohtani *et al*.^49^ reported that fast plasma sheet flows do not reach the geosynchronous orbit or lead to dipolarization. The results of the present study offer an interesting possibility that substorm led injections or BBFs, in general, may not travel all the way to the inner magnetosphere. However, substorm BBFs accompanied by strong convection (*VB*_*south*_) may penetrate the inner magnetosphere and contribute to the ring current.

Our analysis demonstrates the great potential of combining a causal discovery algorithm with a multivariate and lag-specific extension of transfer entropy for tackling contemporary research questions in magnetospheric physics, such as the storm-substorm relationship, which is one of the most controversial topics of magnetospheric dynamics and solar-terrestrial coupling. Further analyses using a causal pathway-analysis^36,50^ can shed light on the interaction mechanism among the solar drivers and the magnetosphere. The obtained causal drivers, on the other hand, can also be relevant for optimal prediction schemes^51^. We expect that our results will contribute to a better understanding of the dynamic processes related to the coupled solar wind - magnetosphere - ionosphere system by fostering a paradigm shift in our perception of the storm-substorm relationship. They may also have a direct impact on magnetosphere modeling and, consequently, space weather forecasting efforts.

## Methods

The algorithm in ref.^19^ for the estimation of the parents of a variable *Y* uses the idea to successively test for conditional independence between *Y*_*t*_ and each possible past driver (including the past of *Y*) conditioned on iteratively more conditions. Thereby, the condition dimension stays as low as possible in every iteration step which helps to alleviate high dimensionality in estimating CMIs. Here we test only the most relevant set of conditions with the highest CMIs in the previous step. The algorithm then is as follows: We first initialize the preliminary parents ${\mathscr{P}}({Y}_{t})=({{\bf{X}}}_{t-1},{{\bf{X}}}_{t-2},\ldots ,{{\bf{X}}}_{t-{\tau }_{{\rm{\max }}}})$ containing the past of all variables (including *Y*). Starting with *p* = 0, we iteratively increase *p* → *p* + 1 in an outer loop and, in an inner loop, test for all variables ${X}_{t-\tau }^{i}$ from ${\mathscr{P}}({Y}_{t})$ whether

4 $$I({X}_{t-\tau }^{i};{Y}_{t}|{{\mathscr{P}}}^{(p)}({Y}_{t}))=0$$

where ${{\mathscr{P}}}^{(p)}({Y}_{t})$ are the *p* strongest parents among ${\mathscr{P}}({Y}_{t})\backslash \{{X}_{t-\tau }^{i}\}$ according to their CMI. If the CMI is zero at some significance level *α* using the test described below, we remove a link from ${\mathscr{P}}({Y}_{t})$ at the end of each *p*-iteration. The algorithm converges if no larger conditioning sets can be tested. We sort ${\mathscr{P}}({Y}_{t})$ after every iteration according to the CMI values.

We use an advanced nearest-neighbor estimator^28,29^ of CMI that is most suitable for variables with a continuous range of values. This estimator has as a parameter the number of nearest-neighbors *k* which determines the size of hyper-cubes around each (high-dimensional) sample point and, therefore, can be viewed as a density smoothing parameter (although it is data-adaptive unlike fixed-bandwidth estimators). For large *k*, the underlying dependencies are strongly smoothed and may not resolve nonlinearities. We tested different values of *k* to verify the robustness of our results. Larger *k* have larger bias and are more computationally expensive, but have smaller variance. Note that the estimated CMI values can be slightly negative while CMI is a non-negative quantity. In Figs. 3 and 4 and Supplementary Figs. S1 and S2 the (C)MI values have been rescaled to the (partial) correlation scale via $I\to \sqrt{1-{e}^{-2I}}\in [0,1]$^27^. In the tables, on the other hand, the CMI values are given in *nats*.

For significance testing, either a fixed threshold or shuffle surrogates are the only choice here. Surrogate tests are especially helpful for proper significance tests because they adapt to the bias for higher-dimensional CMIs. In ref.^17^ a shuffle test has been used, but for strongly autocorrelated time series, as in the present case, this test is too weak. Therefore, we use a block-shuffle surrogate test here following refs.^34^ and^35^. An ensemble of *M* values of $I({X}_{t-\tau }^{\ast };{Y}_{t}|Z)$ is generated where ${X}_{t-\tau }^{\ast }$ is a block-shuffled sample of *X*_*t*−*τ*_, i.e., with blocks of the original time series permuted. As an optimal block-length we use the approach described in refs.^34^ and^35^ for non-overlapping blocks. The optimal block-length (Eq. (6) in ref.^35^) involves the decay rate of the envelope of the autocorrelation function *γ*(*τ*). The latter is estimated up to a maximum delay of 5% of the (non-masked) samples and the envelope was estimated using the Hilbert transform. Then a function *Cϕ*^*τ*^ is fitted to the envelope with constant *C* to obtain the decay rate *ϕ*. Finally, the CMI values are sorted and a *p*-value is obtained as the fraction of surrogates with CMI greater or equal than the estimated CMI value. We use an ensemble of 200 surrogates. Confidence intervals (errorbars in figures) were estimated using bootstrap resampling involving only estimated nearest-neighbor statistics with 200 samples. The block-shuffle approach is only an approximation to obtain the true null distribution.

### Software Availability

Software is available online under https://github.com/jakobrunge/tigramite.

## Electronic supplementary material

Supplementary Material File
