# Supplementary material for: Common solar wind drivers behind magnetic storm–magnetospheric substorm dependency
Source: Sci Rep. 2018 Nov 19;8:16987. doi: 10.1038/s41598-018-35250-5 (PMC6242910; doi:10.1038/s41598-018-35250-5)
Supplement: Supplementary file 1 — Supplementary Material File [file 41598_2018_35250_MOESM1_ESM.pdf]

Supplementary Material

for "Common solar wind drivers behind magnetic storm–magnetospheric substorm dependency"

by Jakob Runge, Georgios Balasis, Ioannis A. Daglis, Constantinos Papadimitriou, and Reik V. Donner

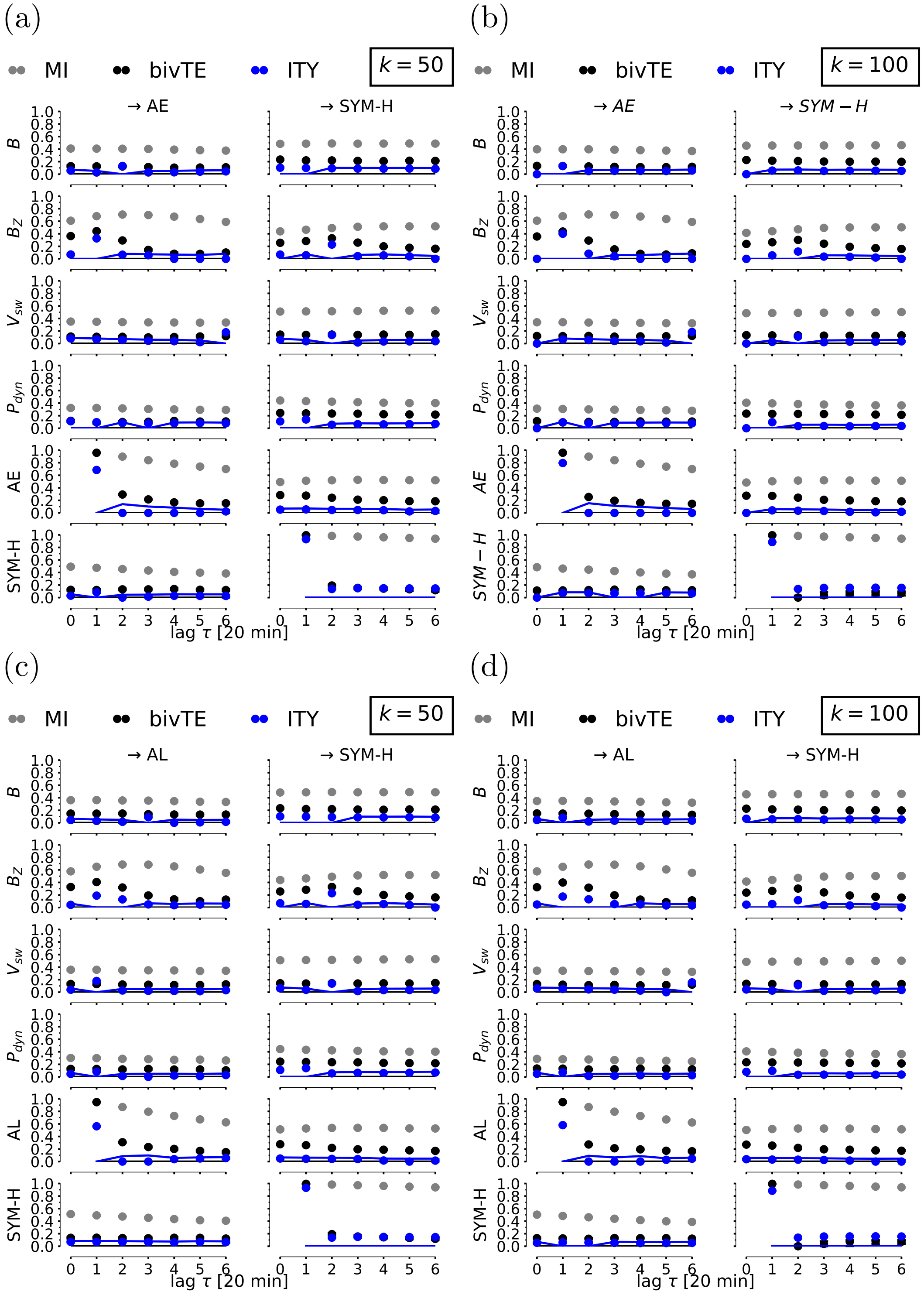

Figure S1. Lag functions as in Fig. 3, but for additional parameters: nearest-neighbor CMI estimation parameter $k = 50$ (a,c) and $k = 100$ (b,d) resulting in a stronger smoothing of the densities; substorm index AE (a,b) and AL (c,d).

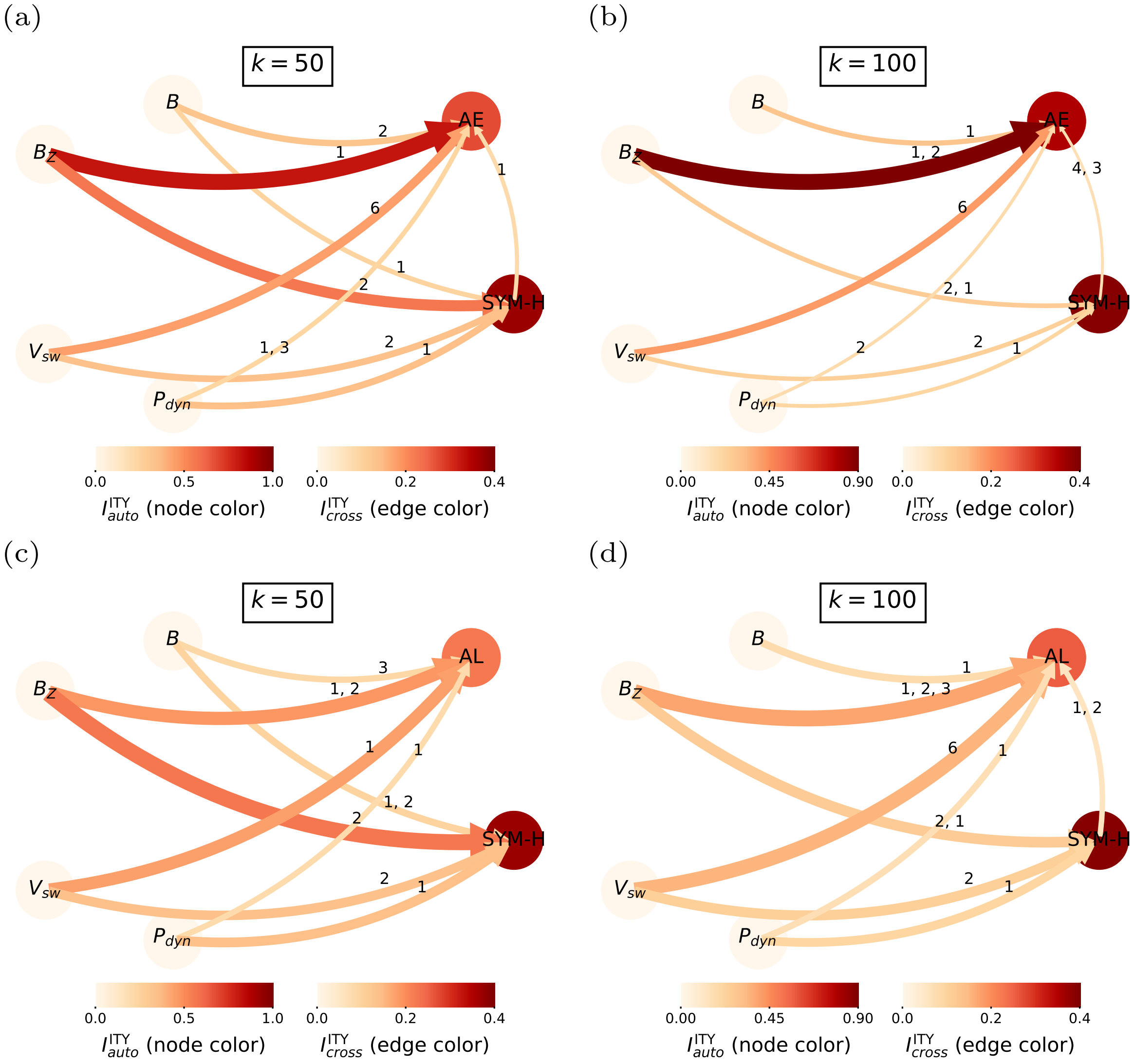

Figure S2. As in Fig. 4, but for additional parameters: nearest-neighbor CMI estimation parameter $k = 50$ (a,c) and $k = 100$ (b,d) resulting in a stronger smoothing of the densities; substorm index AE (a,b) and AL (c,d).

Moderate			Intense			Super-storm		
Date	Time	Dst (nT)	Date	Time	Dst (nT)	Date	Time	Dst (nT)
2001/01/24	07:00:00 PM	-61	2001/03/19	11:00:00 PM	-105	2001/03/31	09:00:00 AM	-387
2001/02/13	10:00:00 PM	-50	2001/03/20	01:00:00 PM	-149	2001/04/11	12:00:00 AM	-271
2001/03/05	05:00:00 AM	-73	2001/04/18	07:00:00 AM	-114	2001/11/06	07:00:00 AM	-292
2001/03/23	05:00:00 PM	-75	2001/04/22	04:00:00 PM	-102	2001/11/24	05:00:00 PM	-221
2001/03/28	04:00:00 PM	-87	2001/08/17	10:00:00 PM	-105			
2001/04/09	07:00:00 AM	-63	2001/09/26	02:00:00 AM	-102			
2001/04/13	04:00:00 PM	-77	2001/10/01	09:00:00 AM	-148			
2001/05/10	02:00:00 AM	-76	2001/10/03	03:00:00 PM	-166			
2001/06/18	09:00:00 AM	-61	2001/10/21	10:00:00 PM	-187			
2001/09/13	08:00:00 AM	-57	2001/10/28	12:00:00 PM	-157			
2001/09/23	07:00:00 PM	-73	2001/11/01	11:00:00 AM	-106			
2001/10/09	04:00:00 PM	-64						
2001/10/11	08:00:00 PM	-70						
2001/10/12	01:00:00 PM	-71						
2001/10/19	10:00:00 PM	-57						
2001/12/21	11:00:00 PM	-67						
2001/12/24	11:00:00 AM	-55						
2001/12/30	06:00:00 AM	-58						

Table S1. Storm classifications for 2001 as a year of strong solar activitiy. We distinguish moderate (-50 nT > minimum of Dst > -100 nT), intense (-100 nT > minimum Dst > -250 nT), and super-storm (minimum of Dst < -250 nT) events.

Parents of SYM-H ($k = 100$)			
Parent (τ [20 min.])	$I_{\mathrm{Par}\rightarrow\mathrm{SYM-H}}$ [nats]	$I_{\mathrm{AL}\rightarrow\mathrm{SYM-H}}$ [nats]	p-value
No conds.		0.1569	$< 10^{-2}$
SYM-H (-1)	2.2162	0.0336	$< 10^{-2}$
+ B_Z (-2)	0.0479	0.0069	$< 10^{-2}$
+ P_{dyn} (-1)	0.0126	0.0018	$< 10^{-2}$
+ V_{sw} (-2)	0.0077	0.0010	0.145
+ B_Z (-1)	0.0016		
Parents of AL ($k = 100$)			
Parent (τ [20 min.])	$I_{\mathrm{Par}\rightarrow\mathrm{AL}}$ [nats]	$I_{\mathrm{SYM-H}\rightarrow\mathrm{AL}}$ [nats]	p-value
No conds.		0.1330	$< 10^{-2}$
AL (-1)	1.1489	0.0079	$< 10^{-2}$
+ B_Z (-1)	0.0865	0.0052	$< 10^{-2}$
+ AL (-4)	0.0125	0.0051	$< 10^{-2}$
+ V_{sw} (-6)	0.0136	0.0025	0.035
+ B_Z (-2)	0.0078	0.0018	0.060
+ B (-1)	0.0045		
+ P_{dyn} (-1)	0.0026		

Table S2. As in Tab. 1, but for a nearest-neighbor CMI estimation parameter $k = 100$, i.e., with a stronger smoothing of the densities.

Parents of SYM-H ($k = 50$)			
Parent (τ [20 min.])	$I_{\mathrm{Par}\to\mathrm{SYM-H}}$	$I_{\mathrm{AE}\to\mathrm{SYM-H}}$	p-value
No conds.		0.1527	$< 10^{-2}$
SYM-H (-1)	2.2092	0.0395	$< 10^{-2}$
+ B_Z (-2)	0.0571	0.0100	$< 10^{-2}$
+ P_{dyn} (-1)	0.0164	0.0032	$< 10^{-2}$
+ V_{sw} (-2)	0.0094	0.0016	0.405
Parents of AE ($k = 50$)			
Parent (τ [20 min.])	$I_{\mathrm{Par}\to\mathrm{AE}}$	$I_{\mathrm{SYM-H}\to\mathrm{AE}}$	p-value
No conds.		0.1281	$< 10^{-2}$
AE (-1)	1.2574	0.0074	$< 10^{-2}$
+ B_Z (-1)	0.1088	0.0074	$< 10^{-2}$
+ B_Z (-3)	0.0093	0.0050	$< 10^{-2}$
+ B (-2)	0.0085	0.0045	0.015
+ V_{sw} (-6)	0.0177	0.0036	0.030
+ SYM-H (-1)	0.0036		

Table S3. As in Tab. 1, but for another substorm index (AE) and $k = 50$.

Parents of SYM-H ($k = 100$)			
Parent (τ [20 min.])	$I_{\text{Par}\to\text{SYM-H}}$	$I_{\text{AE}\to\text{SYM-H}}$	p-value
No conds.		0.1469	$< 10^{-2}$
SYM-H (-1)	2.2162	0.0382	$< 10^{-2}$
+ B_Z (-2)	0.0479	0.0079	$< 10^{-2}$
+ P_{dyn} (-1)	0.0125	0.0024	$< 10^{-2}$
+ V_{sw} (-2)	0.0077	0.0012	0.125
+ B_Z (-1)	0.0016		
Parents of AE ($k = 100$)			
Parent (τ [20 min.])	$I_{\text{Par}\to\text{AE}}$	$I_{\text{SYM-H}\to\text{AE}}$	p-value
No conds.		0.1205	$< 10^{-2}$
AE (-1)	1.2673	0.0067	$< 10^{-2}$
+ B_Z (-1)	0.1056	0.0060	$< 10^{-2}$
+ B (-1)	0.0102	0.0045	$< 10^{-2}$
+ V_{sw} (-6)	0.0174	0.0026	0.080

Table S4. As in Tab. 1, but for another substorm index (AE) and $k = 100$.
